# Intergroup food transfers in wild golden lion tamarins (*Leontopithecus rosalia*)

**DOI:** 10.1007/s10329-020-00846-x

**Published:** 2020-08-07

**Authors:** Camille A. Troisi

**Affiliations:** 1 School of Biology, University of St Andrews, St Andrews, UK; 2 School of Biological, Earth and Environmental Sciences, University College Cork, Cork, Ireland

**Keywords:** Food transfers, Social bond, Intergroup interaction, Golden lion tamarins, Tolerance

## Abstract

The transfer of food between adults is uncommon in primates. Although golden lion tamarins (*Leontopithecus rosalia*) are unique among primates in the extent to which they transfer food, reports of food transfers between adults have so far been restricted to captive or reintroduced individuals. Here, I report the first recorded events of adult–adult food transfers in golden lion tamarins between individuals belonging to different groups in the wild. Given that individuals emigrate from their natal group to find reproductive opportunities, I suggest that intergroup food transfers could be a way for individuals to estimate the quality or availability of potential mates or social partners. I propose an additional function of food transfers in wild golden lion tamarins: that they create and strengthen social bonds with individuals outside of the family group.

## Introduction

Within-group food transfers are common in primates, particularly in apes and callitrichids (Brown et al. 2004), and can serve several functions. Here, the term food transfer refers to any behavioural interaction that involves the passage of food between two individuals, including begging, stealing and offering (see Brown et al. 2004), without implying any intentionality on either side. Most transfers are passive, where the donor allows the receiver to take food from them (Brown et al. 2004), and most occur between mothers and infants (Brown et al. 2004; Feistner and McGrew 1989). Adult-adult food transfer is rare and is only present in species that also transfer food to their young (Jaeggi and Van Schaik 2011). A common function of transfers is hence to provide young with food or information, but between adults, food transfers can be used to avoid harassment (Brown et al. 2004; Feistner and McGrew 1989; Jaeggi and Van Schaik 2011). In support of this second function, most adult–adult food transfers in primates are predicted by dominance, with higher ranking individuals taking food from lower ranking individuals, with relinquishing food being the least costly strategy for the lower ranking individual (Brown et al. 2004). Thirdly, some work on chimpanzees suggests that food transfers between group members are used for social support, or that food is exchanged for sexual interaction (Mitani and Watts 2001; Nishida et al. 1992). Finally, there is also evidence of reciprocal altruism involving food transfers in capuchins and tamarins in captivity (Brown et al. 2004). However, Cheney and Seyfarth (1990) suggest that reciprocity in primates is more likely to involve social interactions rather than physical objects, such as food.

By contrast, between-group food transfers are almost unknown in primates. Recently, Fruth and Hohmann (2018) reported an event where an individual bonobo (*Pan paniscus*) possessing an antelope shared part of it with members of its own group as well as members of the neighbouring community, but comparable observations do not exist for many other commonly studied primates. We might expect to see between-group transfers when there is high tolerance between groups, such as when the cost of aggression is high, or when resources are not defensible (Robinson and Barker 2017).

The Callitrichidae is a unique family not only because of the extensive transfer of food from adults to juveniles, potentially for both nutritional and informational benefits (Brown et al. 2005; Feistner and Chamove 1986; Moura et al. 2010; Moura and Langguth 1999; Rapaport 1999; Troisi 2017; Troisi et al. 2020; Voelkl et al. 2006), but also because of the prevalence of active transfers initiated by adults (Brown et al. 2004; Feistner and McGrew 1989), especially towards juveniles and pregnant females (Guerreiro Martins et al. 2019; Ruiz-Miranda et al. 1999). Recent work has shown that food transfers in callitrichids can also be used to reinforce cooperative bonds within a group (Guerreiro Martins et al. 2019).

The golden lion tamarin (*Leontopithecus rosalia*) is an endangered callitrichid native to the Atlantic forest on the southeastern coast of Brazil. They are territorial cooperative breeders that live in small family groups, with an average group size of five to seven individuals (Dietz et al. 1994; Dietz and Baker 1993; Tardiff et al. 2002; Troisi 2017; Troisi et al. 2018, 2020). Golden lion tamarins have a rapid reproductive turnover, often giving birth to twins, and show intense parental investment (Dietz et al. 1994). They defend a territory of approximately 45.2 ± 15.5 ha against other golden lion tamarin groups (Dietz et al. 1997), and have regular, highly vocal encounters with neighbouring groups (Peres 1989). One study reported that intergroup encounters, including both face-to-face and long-range encounters, occurred on average once every 2.1 and 1.6 days per year of the study, respectively (Peres 1989). Another study reported intergroup interactions in two groups in 11.6% and 2.2% of scan samples, respectively (Dietz et al. 1997). Most golden lion tamarins disperse from their natal groups, mainly by emigrating to neighbouring groups, with 60% of individuals dispersing from their natal group by 3 years of age and 90% after 4 years of age (Baker et al. 2002).

Unlike most primates, golden lion tamarins actively provision young and other group members with solid food (Rapaport and Brown 2008), and adults vocalise to infants to offer them food (Brown and Mack 1978; Rapaport 2011; Rapaport and Ruiz-Miranda 2002). Experimental studies show that golden lion tamarins preferentially transfer to juveniles food items that are rare, that the donors have eaten before, or that are difficult to process or novel (Price and Feistner 1993; Rapaport 1999, 2006; Troisi et al. 2020), but adults also transfer food to pregnant females (Ruiz-Miranda et al. 1999). However, despite food transfers being common, all reported transfers occurred between members of the same group, and transfers between adults have only been reported in captive or reintroduced individuals.

The function of intergroup food transfers still raises a large number of questions. High tolerance between groups or resource defence have been suggested as the driving force behind intergroup food transfers (Robinson and Barker 2017). Interestingly, the social and physical structure of golden lion tamarins’ environment does not predispose them to between-group food transfers, as they are highly territorial and aggressive towards non-group members, and mainly feed on fruits and insects that can be monopolised (Robinson and Barker 2017). Here I describe six events in the wild where food that could be monopolised was transferred (i.e. successfully changed hands) between members of different groups of golden lion tamarin. These interactions between two individuals included individuals offering the food in their possession to another individual, but also events where one individual attempted to obtain a food item from another individual by emitting vocalisations, by reaching out an arm in the direction of the food, or by directly grabbing the food. Given that the previously hypothesised drivers of food transfers between groups have not been found for golden lion tamarins, this is an interesting species to study to further our understanding of the function of intergroup food transfers.

## Materials and methods

Six groups of golden lion tamarins comprising from three to 10 free-living individuals (*n* = 42 individuals in January–March 2014, and *n* = 46 individuals in August–September 2014) were observed in two locations in the Atlantic forest, Brazil. In each group, all of the individuals were related to the breeding pair of that group, with the exception of one individual in three of the groups. Three of the groups were located in the Poço das Antas Biological Reserve (PDA) (22°30′–22°33′S, 42°15′–42°19′W), and the three other groups in a pocket of Atlantic forest in the Fazenda Afetiva-Jorge (FAJ), Imbaú region (22°37′S, 42°28′W). The sites, which are less than 30 km apart, are in the municipality of Silva Jardim, Rio de Janeiro, Brazil, and have similar plant species (Carvalho et al. 2006). Table 1 shows the composition of each group for which observations are reported here (for a list of all the individuals of each group, see Table S1, ESM). The groups were habituated to human presence and monitored by members of the Associação Mico-Leão-Dourado. To keep track of the population, each group was regularly surveyed to record births and deaths, and individuals were captured twice a year to weigh, measure and individually mark them on the tail and body with Nyanzol dye. Individuals were also tattooed at first capture as part of the management of the species by the Associação Mico-Leão-Dourado (Ruiz-Miranda et al. 1999).

**Table 1:** Group composition during the observation period

Group	Location	Male:female	Adults:subadults:juveniles:infants
AF2	Fazenda Afetiva-Jorge	6:6	5:3:2:2
Super	Fazenda Afetiva-Jorge	2:7	3:3:1:2
AF	Poço das Antas Biological Reserve	2:3	4:1:0:0
BO2	Poço das Antas Biological Reserve	5:3	6:2:0:0

Adults are > 18 months old, sub-adults are between 9 and 18 months old, juveniles are between 3 and 9 months old, and infants are < 3 months old

**Fig. 1:**
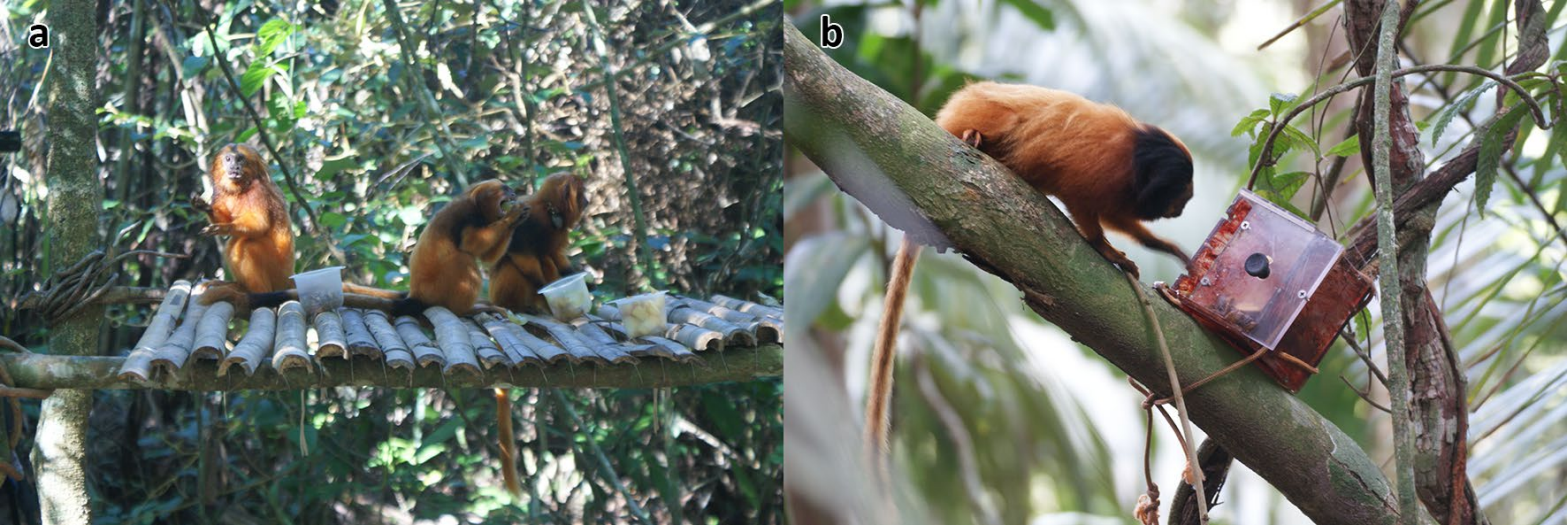
**a** Photo of the food-transfer experiment; **b** photo of the food-offering-call experiment

The observations reported here were made during two experiments designed to study teaching behaviour in golden lion tamarins, during which food was provided to groups in the wild (Troisi et al. 2018, 2020). In the first experiment, golden lion tamarins were provided with small samples of different types of food, i.e. bananas, apples, grapes, mealworms and crickets in January–February 2014, and additionally pears and papayas in August–September 2014 (Troisi et al. 2020). The fruits were cut into small pieces/slices, and the insects were dehydrated. All food items were provided in pots (Fig. 1a). The aim of the experiment was to examine the function of adult to juvenile within-group food transfers. In this experiment, very few individuals ate the dehydrated insects, and all of the transfers observed were of fruits. In the second experiment, the same golden lion tamarin groups were provided with a novel substrate containing slices of bananas in February–March 2014 and September–October 2014 (Troisi et al. 2018) (Fig. 1b), with the aim of determining whether juvenile golden lion tamarins learn substrate properties from food-offering calls (Troisi et al. 2018). In both experiments, territorial encounters took place during some of the trials, allowing me to make the observations that I report below.

All electronic supplementary materials are available at OSF (https://osf.io/8w5gj/).

## Results

I observed six food transfers between individuals of different groups (Table 2; ESM video). All of the transfers reported below involved six unique donors and six unique receivers. One individual (BO2T13) was a donor in one of the observations and a receiver in another observation, thus *n* = 11. In each food transfer, individuals were from different natal groups and unrelated to each other. The first three observations were made during the first experiment investigating food transfers using different types of food (Troisi et al. 2020), and the remaining three observations during the second experiment, which examined the function of food-offering calls (Troisi et al. 2018).

**Table 2:** Summary of the successful transfers between groups

Obs.^a^	Location	Date	Donor^b^	Donor group	Donor sex	Donor age class	Receiver^c^	Receiver group	Receiver sex	Receiver age class	Resistance during transfer^d^	Resistance at point of transfer^e^
1	FAJ	1 February 2014	AF2T3	AF2	Male	Sub-adult	SuperT3	Super	Female	Sub-adult	Yes	No
2	FAJ	1 February 2014	AF2T1	AF2	Male	Adult	SuperT13	Super	Female	Sub-adult	Yes	No
3	PDA	6 September 2014	BO2T13	BO2	Male	Adult	AFT3	AF	Female	Sub-adult	No	No
4	FAJ	19 February 2014	AF2T34	AF2	Female	Sub-adult	SuperT0C	Super	Male	Adult	Yes	Yes
5	PDA	14 September 2014	AFT234	AF	Female	Adult	BO2T3	BO2	Female	Sub-adult	NA	NA
6	PDA	14 September 2014	AFT0C	AF	Male	Adult	BO2T13	BO2	Male	Adult	NA	NA

Adults are > 18 months old, sub-adults are between 9 and 18 months old

*FAJ* Fazenda Afetiva-Jorge, *PDA* Poço das Antas Biological Reserve

^a^Observations (*Obs*.) are in the same order as listed in this paper

^b^Individual that initially had the food

^c^Individual that obtained the food

^d^Donor resists food transfer up to, but not including, the point of transfer

^e^Donor resists food transfer at the point of transfer

A total of 128 experimental trials were carried out on the study population during the two experiments reported in Troisi et al. (2018, 2020). An individual from another group was observed during 17 of these experimental trials of a focal group. These 17 trials included the six observations of intergroup food transfer (from four different trials) reported here. In addition, the focal group was displaced by another group in two of the trials. Hence, an intergroup encounter was recorded in 14.8% (19/128) of the total number of trials.

### Observation 1

The first observation was made on 1 February 2014 at Fazenda Affetiva-Jorge. Five individuals, four from group AF2 and one from group Super, were foraging on provisioned fruits at a platform before briefly dispersing. The following individuals from group AF2 were present: AF2T3, a sub-adult male; AF2T13, a juvenile male; AF2T2, a juvenile female; and AF2T34 a sub-adult female. The individual from group Super was SuperT3, a sub-adult female (1 year 3 months old). After dispersal to branches around the platform, SuperT3 approached individual AF2T3, who was foraging on a piece of grape. SuperT3 extended her arm and hand four times towards AF2T3 and vocalised before AF2T3 appeared to let her take the food from his hand.

### Observation 2

The second observation occurred 21:48 min after the first one. Two individuals from AF2 (AF2T2, a juvenile female; and AF2T14, an adult male) and one individual from Super (SuperT3, from observation 1) were foraging on a platform when they were approached by individual SuperT13, a sub-adult female (1 year 3 months old) from group Super. AF2T12, an adult female carrying two infants, and AF2T23, an adult female, were also in the vicinity of the platform. SuperT13 first inspected some of the food on the platform before approaching AF2T14, then first attempted to take a grape from AF2T14’s mouth. AF2T14 showed some resistance to this, but then appeared to let SuperT13 take the food from his hand. SuperT13 was then approached by a juvenile from the AF2 group, potentially to take the food, which led SuperT13 to leave the platform. During this entire period, SuperT3 was foraging on another food patch less than 15 cm away from where the food transfer took place.

### Observation 3

The third observation took place on 6 September 2014 at the Poço das Antas Biological Reserve. AFT3, a sub-adult female from group AF, was foraging in the presence of two other individuals (AFT234, an adult female from group AF, and an unidentified individual, potentially also from group AF), when BO2T13, an adult male from group BO2, approached the pot of food where AFT3 (11 months old) was foraging. BO2T13 started extracting a piece of grape from the pot, but AFT3 took it from BO2T13’s hands and ate it, with little resistance from BO2T13. During the transfer, BO2T2, a sub-adult male from group BO2, was in the vicinity of the pot.

### Observation 4

This observation, and the following two, were made during the food-offering call experiment (Troisi et al. 2018) where a novel substrate containing banana was provided to the groups. On 19 February 2014 at Fazenda Affetiva-Jorge, three individuals were foraging on the novel substrate: one from group Super (SuperT0C, an adult male) and two from group AF2 (AF2T4, a sub-adult male; and AF2T34, a sub-adult female). AF2T34 obtained food from the substrate and was eating some banana when SuperT0C (3 years 4 months old) arrived to investigate the substrate, then tried to get some banana from AF2T34’s hands. AF2T34 showed some resistance, but SuperT0C obtained the food nonetheless. SuperT3, a sub-adult female, and SuperT1, a juvenile female, both from group Super, approached the group during the transfer.

### Observation 5

The firth observation occurred on 14 September 2014 at the Poço das Antas Biological Reserve. Four individuals from group AF were present: AFT3, a sub-adult female; AFT0C, an adult male; AFT234, an adult female; and AFT1234, an adult female; as well as one individual from group BO2, BO2T3, a sub-adult female. AFT234 was extracting food from the novel substrate when BO2T3 (12 months old) intercepted the food and obtained part of the slice of banana that AFT234 had been trying to extract. BO2T3 actually attempted to get food from AFT0C, an adult male from group AF, and from AFT3, a sub-adult female from group AF, in the 12 s preceding the successful transfer from AFT234. However, both of these former attempts were unsuccessful (i.e. BO2T3 did not obtain any food).

### Observation 6

The sixth observation occurred 15 s after the fifth one. AFT1234, AFT0C and AFT234 from group AF were foraging at the platform, and BO2T3, from group BO2, was eating the transferred piece of banana nearby, when BO2T13, an adult male (1 year 7 months old) from group BO2 approached the substrate. AFT0C was extracting food from the substrate when BO2T13 reached up from below and grabbed the piece of banana before AFT0C could put it in his mouth. BO2T13 left the area immediately after having obtained the food.

In the first three observations the individual who initially had the food stopped resisting before the second individual obtained the food. In each of these transfers the individual who initially had the food was male and the individual who obtained it was female. Two of the donor males were adults (> 18 months old), and one was a subadult (between 10 and 18 months old), while all three of the receiver females were subadults. In the fourth observation, where the food was transferred from a sub-adult female to an adult male, the adult male received the food despite the sub-adult female resisting at the point of transfer. In the last two observations, in which the food was transferred from an adult female to a sub-adult female (fifth observation), and from an adult male to another adult male (sixth observation), the transfer of food took place quickly, and I was unable to see whether there was any resistance during the transfer.

## Discussion

The six observations reported here show that the transfer of food between strangers is not unique to humans or other great apes. In each case, the food items that were transferred could have been obtained individually, without any requirement for cooperation or specialised skills. This is particularly true for observations 1–3 as food was available in several areas other than the place where the food transfer occurred. In these three cases, individuals could have easily taken food directly from the pots, and even sometimes from individuals of their own group. In observations 4–6, however, there was only one source of food present, leading to congregation around the device. This lack of multiple food sources in observations 4–6 could have meant that it was easier for an individual to obtain food from another individual, even if that individual was from another group, than obtain food from the device, despite the agonistic interaction that could have arisen as a consequence of this. However, these six food transfer events were very similar to those that occur between individuals of the same group.

These instances of transfer of food between members of different groups of golden lion tamarin may show, for the first time, a level of tolerance in this species similar to that seen in bonobos (Fruth and Hohmann 2018). However, the social structures of golden lion tamarins and bonobos are very different: while bonobos show moderate aggression towards other individuals, including individuals of other communities, and live in fission–fusion groups within a community, golden lion tamarins are aggressive towards potential immigrants, and live in a family-structured group where offspring emigrate, on average, when they are around 2.5 years old (Baker and Dietz 1996; Fruth and Hohmann 2018; Romano et al. 2019). Moreover, unlike in bonobos, the food resources that were transferred between the golden lion tamarins could have been easily monopolised by just one individual. Both the social and physical structure of golden lion tamarins’ environment are very different to those of other primate species, such as bonobos, in which intergroup food transfers have been observed. It is therefore unlikely that tolerance between different groups or resource defence help explain intergroup food transfers in golden lion tamarins, and likely that this type of sharing between groups evolved independently in different primate linages. In primates, there is evidence that food transfers may be used by females to test a male’s tolerance (Goffe and Fischer 2016; van Noordwijk and van Schaik 2009; Yamamoto 2015). Yamamoto (2015) proposed a begging-for-social-bond hypothesis, in which individuals beg to strengthen social bonding as well as to gain access to food. Although insufficient, if supported by further experiments, the observations described here would indicate that this hypothesis is applicable to species other than those of great apes, and also that social bonds may extend beyond a group. Given that the observations of golden lion tamarins reported here violate previous expectations of which social and environmental conditions drive food transfers between groups, additional work on this species could further our understanding of the function of intergroup food transfers.

Although food transfers between different groups of golden lion tamarins could be indicative of a new level of social tolerance in this species, it is important to note that they are highly territorial, with intergroup interactions usually being aggressive (French and Inglett 1989; Peres 1989; Ruiz-Miranda et al. 2002). During dispersal events, resident golden lion tamarins are also aggressive toward immigrants (Baker and Dietz 1996), so the six observations of food transfer between individuals of different groups reported here are particularly interesting, and also inconsistent with previously suggested functions of food transfer in this species. In golden lion tamarins, food transfers have mainly been studied in the context of providing nutrition or information to juveniles, or nutrition to pregnant females. Here I suggest that food transfers may be used to create and/or strengthen social bonds with non-group members.

Subordinate golden lion tamarins have two main reproductive options: to wait for a breeding opportunity in their natal group, while caring for the young of the breeding pair, or to emigrate to explore their own breeding opportunities (Romano et al. 2019). Both male and female golden lion tamarins disperse from their natal group and settle in the first available breeding position or unoccupied area that they encounter; however, males tend to disperse more frequently than females, and are more successful when dispersing (Baker and Dietz 1996; Dietz and Baker 1993; Moraes et al. 2018; Romano et al. 2019). Furthermore, males and females use different strategies to emigrate: males are more likely to immigrate into established groups, whereas females are more likely to form new groups (Romano et al. 2019), and are also more likely to inherit their natal territory than males (Baker and Dietz 1996). Leaving the natal group for reproduction is very risky for tamarins, but encounters with neighbouring groups provide opportunities to identify potential sexual partners or new group members (Nascimento et al. 2014). Food transfers between individuals of different groups could therefore be used to create a social bond prior to immigration, which could either facilitate acceptance and reduce aggression when immigrating to a new group, or enable individuals to find social partners to form a new group with. Work on captive callitrichids suggests that immigration might be limited by aggression from resident individuals, with female residents being particularly intolerant of other females (e.g., French and Inglett 1989; French and Snowdon 1981; Harrison and Tardif 1989). Food transfers with members of a different group could therefore be a potential mechanism to recruit new members into a group by modulating the level of tolerance towards unfamiliar conspecifics (French and Inglett 1989). Food transfers with individuals from a different group, particularly with individuals of a different sex, might enable future immigrants to assess their likelihood of being integrated into a new group, or of finding a mate. Miller et al. (2003) found evidence that scent markings are not used for territorial defence in golden lion tamarins, but might be used as a way to communicate information for mate selection, extra-group copulation, and/or to attract immigrant partners. Food transfers could similarly be used to decrease aggression in order to communicate information beyond the group. If food transfers are indeed used as a means of creating a social bond with non-group individuals prior to dispersal, one would expect individuals involved in between-group food transfers to be of an age to disperse. According to Romano et al. (2019) natal emigration occurs around 2.5 years of age (range: 12–70 months). All the receivers of the between-group transfers in the six reported observations were within this range, except for one tamarin which was 11 months old, and all the receivers were younger than 2.5 years old, except for one which was 3 years 4 months old. The age of the donors was more variable, i.e. from 1 year 4 months to 7 years 4 months, but all the donors were within the age range given by Romano et al. (2019) except for one individual. However, we would not necessarily expect the age of a donor to fall within the natal emigration age range, as the receiver might be immigrating into the donor’s group, whereas the donor might not necessarily emigrate.

Romano et al. (2019) found that conspecific attraction, where individuals leave their natal group because they are attracted to potential extra-group mates and/or emigrating group mates, characterises emigration for both male and female golden lion tamarins. Food transfers might be a way of assessing potential extra-group mate quality or acceptance, and Hankerson and Dietz (2014) suggest that males in particular might prospect neighbouring groups for breeding opportunities. Hence food transfers might be particularly useful for males deciding where and when to immigrate to reduce the probability of eviction. Romano et al. (2019) also found evidence for parallel dispersal (emigration with peers or close kin) in golden lion tamarins. Since females are more likely to start new groups than males, they might evaluate potential mates or social partners for this through intergroup food transfers. Long-term data are required to determine whether individuals involved in food transfers with members of other groups then immigrate preferentially into these groups, or start new groups with members who have shown tolerance towards them.

Four out of the six observations reported here were food transfers between individuals of different sex: in three observations (1, 2, 3) the food went from a male to a female, while in one observation (4) it went from a female to a male. These four observations might be examples of individuals assessing the quality of a potential mate through food transfer prior to dispersing. If so, these observations would support the sexual selection hypothesis, which postulates the occurrence of competition in the choice of a mate (West-Eberhard 1983). However, two of the observations were of food transfer between individuals of the same sex: in observation 5 the food went from a female to a female, and in observation 6 the food went from a male to a male. It is possible that instead of being a means of helping a tamarin to choose between potential mates, these food transfer events also help individuals to select a future social partner prior to dispersing. It is also interesting to note that the two food transfers between individuals of the same sex took place rapidly, and were more akin to what is described in the literature as “food stealing”. Overall, I suggest that food transfer to a member of a different group may be a means of creating a social bond with that individual, especially prior to dispersal. Further data are required to assess which of these outcomes is more likely to primarily drive food transfers between adults of different groups.

One limitation of the observations reported here is that they were made during an experiment where food items were provided to golden lion tamarin groups. Thus it is possible that the increase in food availability induced an atypical level of tolerance towards non-group members, resulting in the observed food transfers. However, I think it is unlikely that the increase in food availability created this high level of tolerance because half of the observations (3, 5, and 6) were made during the fruiting season, when so much food was available that it was sometimes difficult to interest the groups in the fruit provided in the experiments, as they were more interested in the fruit in the trees. Furthermore, some experiments undertaken on tamarins in captivity have indicated that food transfers are less likely when food is abundant (e.g. Price and Feistner 1993) because it is easier for the tamarins to acquire food personally instead of getting it from another individual. Moreover, although interactions between different groups are usually quite aggressive initially, they can last several hours, and lose intensity over time (Peres 1989). During face-to-face encounters, when no experiments are taking place, individual golden lion tamarins still forage, although less than at other times (Peres 1989). When a group’s territory overlaps with that of another group and when encounters de-escalate, individuals from different groups have been observed feeding on the same tree (C. R. Ruiz-Miranda, personal communication). Although not widely reported in the literature, this is relatively common, particularly in large ficus trees (C. R. Ruiz-Miranda, personal communication). Juveniles from different groups have also been observed playing together, without much interference from adults (C. R. Ruiz-Miranda, personal communication), suggesting a certain level of tolerance, at least towards some individuals. Hence, given those observations, it seems probable that food transfers between individuals of different groups occur outside of the context of human provisioning.

### Conclusion

Most previous work on food transfers in golden lion tamarins has focussed on transfers from adults to young to determine whether the function of the transfer is to provide nutrition, information or both (Price and Feistner 1993; Rapaport 1999; Troisi et al. 2020). Up until now, adult-adult food transfers have only been reported in captive or reintroduced individuals (Ruiz-Miranda et al. 1999). Here there is not only evidence of adult-adult food transfers in the wild, but also of food transfers between individuals of different groups, which is inconsistent with the previously suggested functions of food transfers in this species. I suggest an additional function of food transfers in wild golden lion tamarins: that they create and/or strengthen social bonds with individuals outside of the family group, which could be particularly useful for tamarins prior to immigrating to a new group or founding a new group with individuals from other groups. Although the function of intergroup encounters in lion tamarins is not fully understood, the six observations reported here contribute to the growing body of literature showing the flexibility of social behaviour in callitrichids. Taken together, the different functions of food transfers in wild populations of primates offer us insights into their social behaviour (Goffe and Fischer 2016).
